# Direct penetrating and indirect neck trauma as a cause of internal carotid artery thrombosis and secondary ischemic stroke

**DOI:** 10.1007/s11239-014-1077-2

**Published:** 2014-04-20

**Authors:** Karol Karnecki, Zbigniew Jankowski, Michał Kaliszan

**Affiliations:** Department of Forensic Medicine, Medical University of Gdańsk, ul. Dębowa 23, 80-204 Gdańsk, Poland

**Keywords:** Carotid artery injuries, Carotid artery thrombosis, Stroke, Head and neck trauma

## Abstract

The following manuscript presents two cases of ischemic stroke secondary to traumatic internal carotid artery thrombosis with concomitant middle cerebral artery thrombosis occurring very rarely in the medico-legal practice. Penetrating neck trauma due to an occupational accident and multiple head and neck trauma secondary to battery were described. The autopsy and histopathological examination as well as the analysis of available medical records, including radiological examinations, and records of investigation indicated the sustained trauma to be the cause of the thrombosis.

## Introduction

A stroke is defined by the WHO as “rapidly developing clinical signs of a focal (or global) disturbance of cerebral function, with symptoms lasting 24 h or longer or leading to death, with no apparent cause other than of vascular origin” [1].

Taking into consideration the cause, the mechanism and the character of morphological lesions, there are two types of stroke: ischemic and haemorrhagic [2, 3]. Eighty percent of strokes are of ischemic origin [2, 4], and their most common cause is thrombosis of the atherosclerotic internal carotid artery (ICA) and/or cerebral arteries (CA)—20 % [4].

A stroke, including an ischemic one, is of particular interest to the forensic pathologist aiming on establishing the cause of death and its mechanism, rather than for giving an opinion in cases of suspected medical malpractice.

Neck and/or head trauma may very rarely constitute a cause of thrombosis of the ICA and CA without any pre-existing pathology [5–8]. It most commonly results from direct, penetrating or non-penetrating (blunt) neck trauma in the region of large cervical vessels; less frequently it is attributed to indirect head trauma. In such cases the autopsy result and available clinical information has to allow the forensic pathologist to prove the existence of neck and/or head trauma and establish its causal connection with ICA thrombosis and exclude other, known, non-traumatic causes of the latter.

We present two cases of ICA thrombosis with concomitant middle cerebral artery (MCA) thrombosis secondary to the thrombus present in the ICA lumen, in which the postmortem examination, the medical records regarding hospitalization and records of investigation indicated trauma as the cause of thrombosis.

## Case 1

**Fig. 1:**
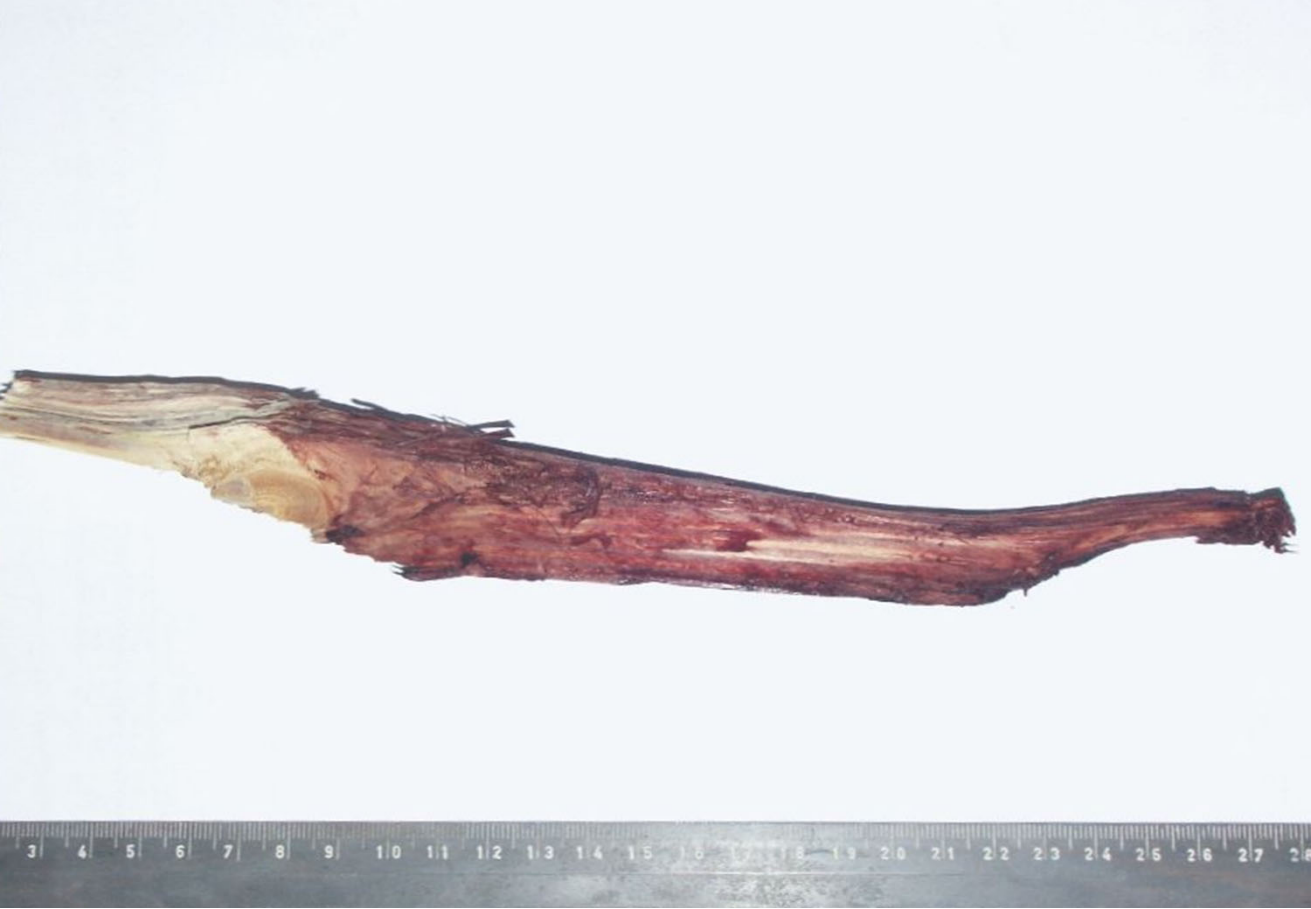
Case 1: “wooden splinter” removed during the surgical procedure

**Fig. 2:**
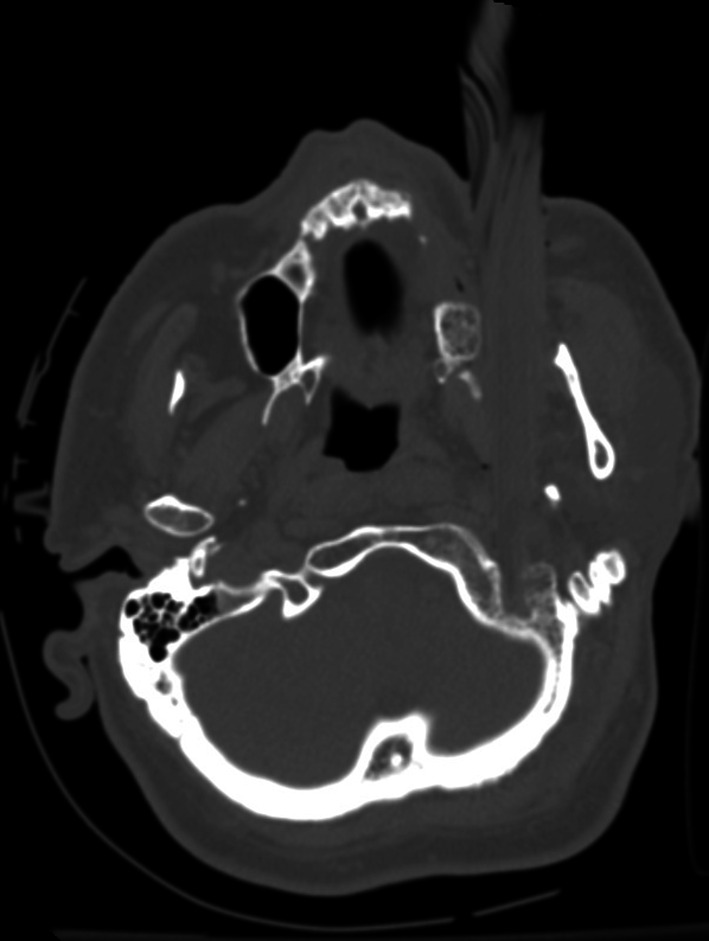
Case 1: localisation of the foreign body (CT)

A 57-year old male carpenter sustained an injury in an occupational accident in the carpenter’s warehouse. He was struck very hard in the face, in the region of his left cheek by an irregular-shaped wood fragment which looked like a big splinter (measuring about 26 cm in length, and with the greatest cross-sectional dimensions of 3.5 × 1.5 cm) that broke off from the board being processed on the carpenter’s machine (Fig. 1). First aid was provided in the emergency department in the local hospital, where the presence of a large, wooden fragment that thrust deep into the left cheek and penetrated through the parapharyngeal space into the neck was diagnosed. On admission the patient was conscious, responsive and oriented. He was then transferred to the otolaryngological ward, where a physical examination, including an evaluation of his neurological status revealed: maintained consciousness, narrow and symmetric pupils, circulatory and respiratory sufficiency and BP of 180/80 mmHg. The CT examination revealed the presence of a foreign body penetrating the left maxillary sinus with a comminuted fracture of the anterior and inferior wall below the external surface of the base of the skull, and a left occipital condyle fracture with concomitant breaking and impression into the posterior cranial fossa and soft tissues of the neck. No cerebral lesions, including traumatic, or features of raised intracranial pressure were observed (Fig. 2). The CT angiography allowed the diagnosis of an occlusion of the ICA by a thrombus localised at the level of the bifurcation of the common carotid artery (CCA) and at the level of C1, adjacent to the foreign body. The laboratory findings were as follows: d-dimer 20921.25 µg/L (*N* < 500); PT and aPTT within normal limits. Once the diagnostic procedures were finished, a surgical intervention was performed on the day of admission. The preparation of the internal jugular vein revealed the medial translocation and compression of the carotid vessels. The foreign body was removed. Post-interventional CT revealed the thrombotic occlusion of the left ICA and MCA and a hypodense area supplied by the MCA and the concomitant left hemisphere oedema of the brain. The patient died on the 6th post-operative day exhibiting symptoms of intracranial hypertension and a brain oedema.

The findings of the medico-legal autopsy were as follows: a thrombus occluding the left ICA and the proximal segment of the left MCA, a large infarction area in the temporal lobe and adjacent parts of the frontal and occipital lobes of the left hemisphere of the brain, a large brain oedema with features of subfalcine herniation and transtentorian herniation, a comminuted fracture of the left maxilla and an occipital bone fracture in the region of the occipital condyle with the fragment impression. There were no atherosclerotic lesions in the carotid and basal CA.

**Fig. 3:**
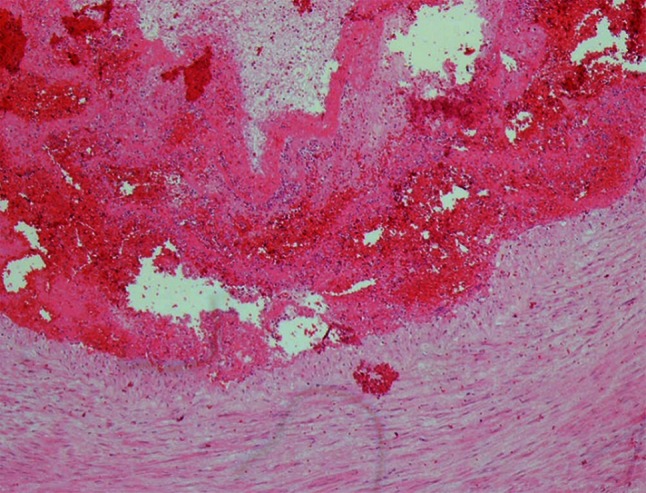
Case 1: the internal carotid artery wall with the adjacent thrombus (H + E)

**Fig. 4:**
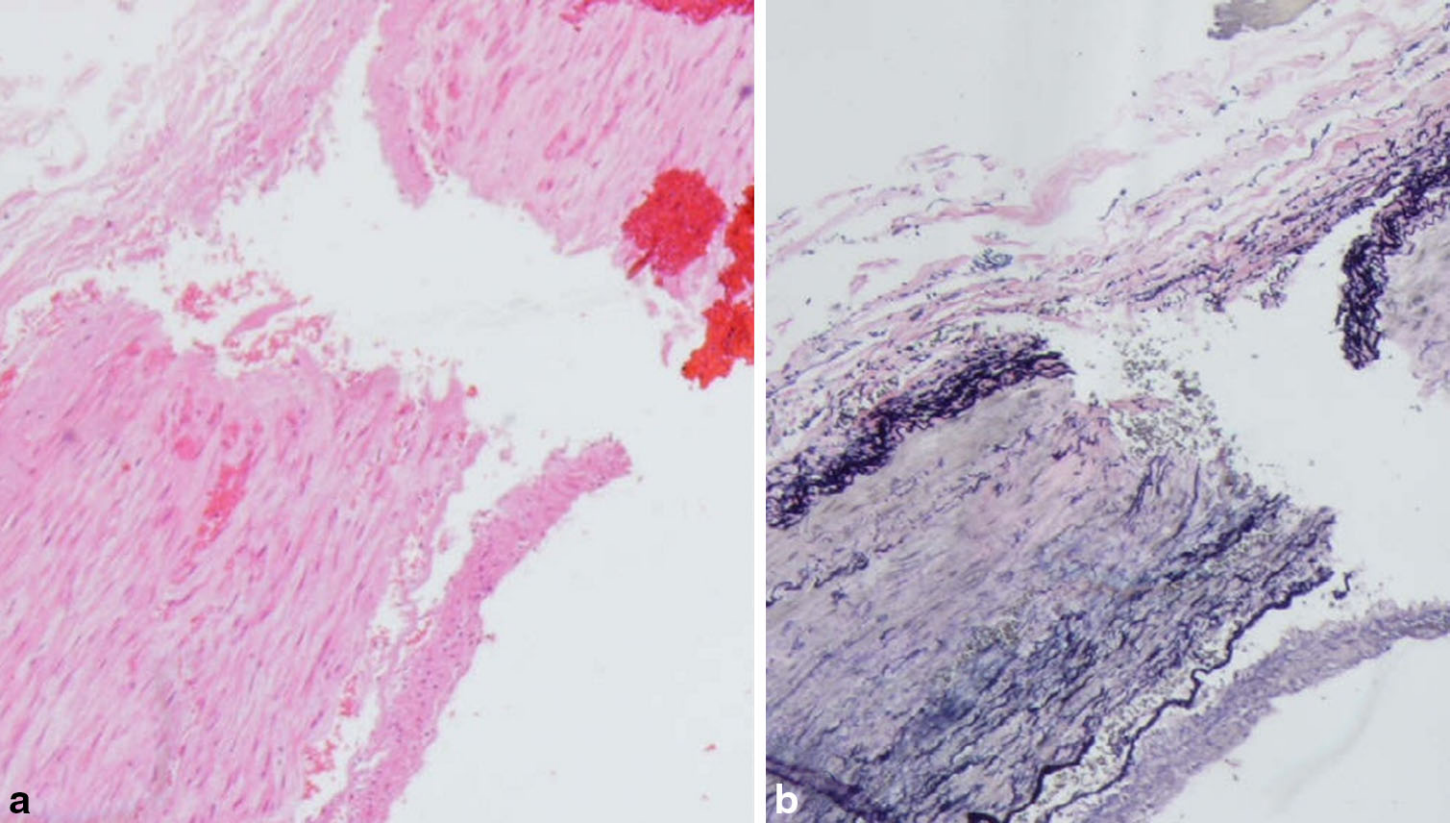
Case 1: damaged tunica media, damaged and dissected intima, as well as fragments of the thrombus within the internal carotid artery (**a** H + E and **b** Verhoeff)

The histopathological examination revealed the occlusion of the left ICA by the thrombus (H + E (Fig. 3), Masson, Gomori, Verhoeff and PTAH), focal intimal haemorrhages and features of a mechanical injury—the rupture of the artery wall in the form of an irregular fissure in the tunica media (Fig. 4). The samples of the macroscopically changed left cerebral hemisphere revealed ischemic necrosis with polymorphonuclear leukocytes reaction, oedema and hyperaemia, while secondary, focal, non-reactive perivascular haemorrhages were found in the brainstem. Other organs showed no significant histopathological lesions, apart from morphological indices of circulatory disturbances.

## Case 2

**Fig. 5:**
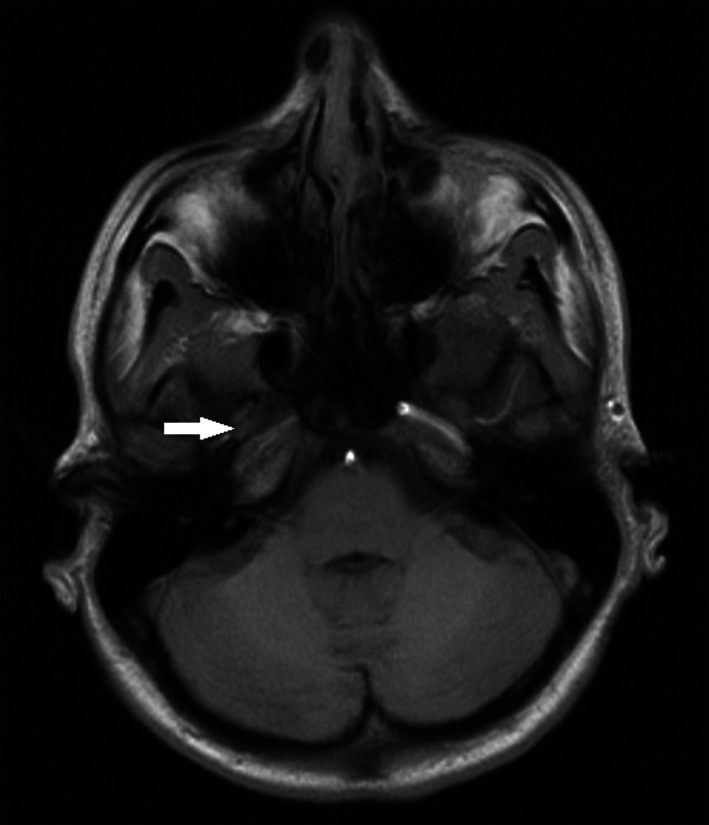
Case 2: occlusion of the right internal carotid and middle cerebral artery marked with an *arrow* (MRI)

**Fig. 6:**
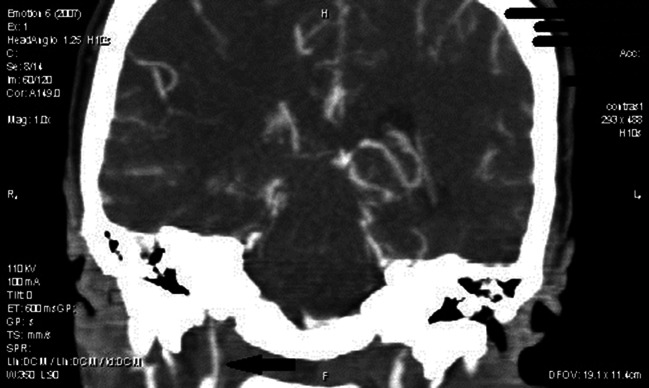
Case 2: occlusion of the right internal carotid artery marked with an *arrow* (angioCT)

A 32-year old female patient was brought to the hospital after being battered what resulted in police intervention. On admission the patient was conscious, complaining about a left upper extremity contracture that started 2 weeks prior to admission. She was generally healthy, with no chronic diseases, taking no medication, apart from being a smoker. For the previous month she had been repeatedly battered by her partner (“about twice a week”) with a fist and open hand to the face, neck and back, as well as pulled by the hair. The last battery that took place 1 day prior to admission resulted in a short-term loss of consciousness. On admission the following body injuries were described in her medical record: resorbing bruises on the face, including a wound of the lower lip mucosa, and the upper extremities,. On admission the patient was sleepy with left-sided hemiparesis with symptoms concerning mainly the upper extremity, blood pressure was 110/80 mmHg, a fundus examination revealed no pathological lesions. A basic laboratory examination of blood and urine was normal. A cerebrospinal fluid examination was normal. d-dimer 1 200 ng/mL (*N* < 500), aPTT slightly shortened 25.69 s (*N*: 28–36), aPTT ratio and PT within normal limits. CT revealed the presence of a hypodense area in the right hemisphere region supplied by the MCA, whereas MRI scan confirmed the presence of an ischemic brain damage. Thrombotic occlusion of the ICA and MCA was visible in MRI (Fig. 5) and angioCT (Fig. 6). Despite intensive therapy, the patient died on the 12th day after admission presenting symptoms of raised intracranial pressure with a concomitant brainstem injury secondary to a brain oedema.

The findings of the medico-legal autopsy were as follows: a thrombus occluding the right ICA and the MCA, thrombosis of the internal jugular veins, as well as the superior sagittal sinus and transverse sinus, an extensive infarction area in the right hemisphere region supplied by the MCA; a brain oedema with features of right-sided subfalcine herniation and transtentorian herniation, carotid and basal CA with no atherosclerotic lesions.

**Fig. 7:**
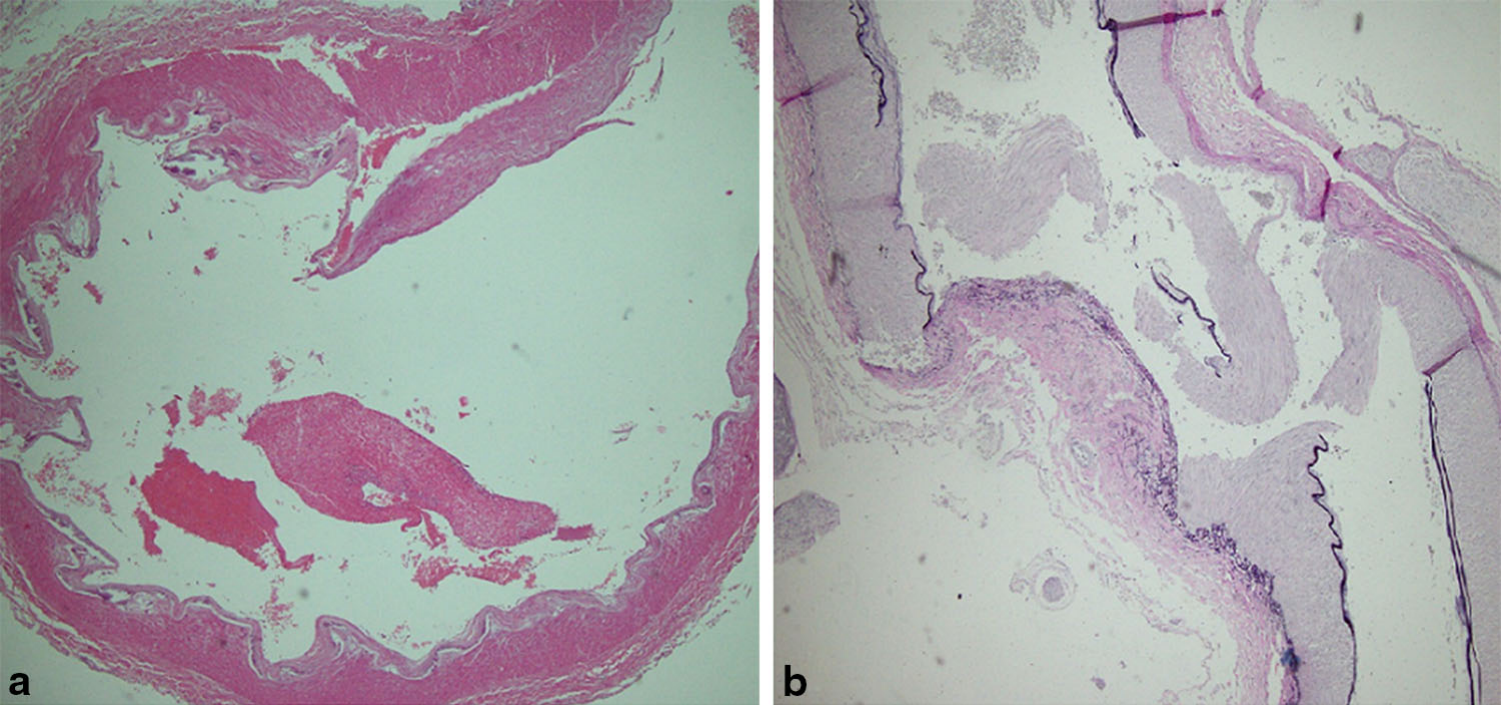
Case 2: damaged tunica media and intima of the internal carotid artery with intact adventitia (**a** H + E and **b** Verhoeff)

A histopathological examination revealed an occlusion of the right ICA by the thrombus (H + E, Masson, Gomori, Verhoeff and PTAH)—a muscular type artery with normal wall structure, the lumen filled with thrombus fragments, mechanical injury of the arterial wall—rupture of the intima and tunica media (Fig. 7). Internal jugular veins—normal wall structure, occluded by thrombi. There were dominating circulatory disturbances in the brain in the form of very significant capillary-venous hyperaemia resulting from an impaired cerebral venous return secondary to jugular vein and dural sinuses thrombosis.

## Discussion

Observed in both cases, focal ischemic brain injury was secondary to the thrombosis of the ICA and MCA.

Cerebral vascular lesions may be causally associated with the sustained head and/or neck trauma with concomitant ICA thrombosis and ischemic brain damage [5, 7–13]. They can, however, accompany head and/or neck trauma, yet be independent of trauma and have other morbid origins [2, 4].

In both cases we considered the most common, known, possible non-traumatic causes for ICA thrombosis, including atherosclerosis, which is the most common cause for ICA and CA thrombosis. A thrombus is formed on the surface of the unstable atherosclerotic plaque. The endothelium and the connective tissue cap injury expose the blood coagulation activating factors, which induce thrombus formation. The endothelium injury itself with exposure of the basal membrane may predispose to thrombus formation as well. The presence of stable atherosclerotic plaque may be complicated by arterial thrombosis, and this refers to cerebral vessels as well. However, vessel stenosis has to be significant, i.e. the blood flow transforms from being laminar into post-stenotic turbulent [14–17]

Both the in vivo neuroradiologic diagnostic imaging modalities, including CT angiography, and the postmortem examination with a histopathological assessment did not reveal the atherosclerosis of ICA or MCA in any of the discussed cases.

Other factors favouring carotid arteries thrombosis include: hypertension, diabetes, hypercholesterolaemia, obesity and smoking [2, 4, 18]. Such factors accelerate the course of atherosclerosis, of which a common complication is thrombosis. The analysis of both patients’ medical records, including the laboratory findings obtained during hospitalisation allowed diabetes and lipid metabolism disorders to be ruled out. The woman did not manifest features of hypertension, whereas unstable aterial pressure values, i.e. labile hypertension, was observed in the male patient. No lesions in the small-sized cerebral vessels that could have been responsible for the ischemic stroke were found during the postmortem examination, including the neuropathologic examination, within the ischemic, as well as in the non-ischemic areas. No arteritis, amyloid angiopathy, vascular malformations nor lesions observed in the course of hypertensive encephalopathy were found.

The presence of a thrombus inside the ICA and/or CA, and secondary focal ischemic brain damage may result from embolism [2, 4, 18, 19]. The most common source of such embolic material are thrombi localised in the left side of the heart (auricle of the left atrium in patients with atrial fibrillation), parietal thrombi in the left ventricle whose formation is associated with myocardial infarction or more frequently aneurysm of the heart in the area of an extensive post-infarction scar or left ventricle dilatation, e.g. in the course of dilated cardiomyopathy or endocarditis, especially of bacterial origin. The emboli may as well result from a so-called crossed embolism. The embolism in the ICA and CA may then develop in patients with atrial septal defect and deep vein thrombosis. The postmortem examination did not, however, reveal any of these conditions in the discussed patients.

A significantly less common cause of stroke are haematological disorders, which are responsible for about 1 % of all strokes and occur slightly more frequently in young subjects. This concerns both ischemic, as well as haemorrhagic strokes [15]. The laboratory findings performed during hospitalisation and autopsies allowed haematological disorders to be ruled out as factors predisposing to thrombosis. The elevation of d-dimer level was the sole abnormal parameter observed on the coagulation profile and was associated with present ICA and MCA thrombosis in both cases. The remaining coagulation parameters were within normal limits.

Oral contraceptives (OC) should be considered as a possible cause for thrombosis in the female patient [20]. OC are described in neurology as one of the ethological factors of ICA and CA thrombosis. In the presented case the anamnesis collected on the patient’s admission to the hospital indicated that she did not take any medication, most probably including OC. However, should she have taken OC, they did not significantly influence the development of ICA thrombosis. Arterial thrombosis in such cases is very rare in women below 35 years of age [21, 22]. The annual incidence of ischemic stroke in women in this age group is 1–3 cases/100 000 women [20]. The following risk factors are considered to increase the risk of developing thrombosis in subjects using OC: hypertension, diabetes, hyperlipidemia and smoking [20–25]. OC predispose more strongly to the development of venous, rather then arterial thrombosis, which results from the haemodynamic properties of blood flow in these two types of vessels [21]. Other risk factors for venous thrombosis are slowed blood flow and thrombophilia, whereas vessel wall injury (endothelium) [23], including the atherosclerotic plaque are characteristic risk factors for arterial thrombosis. Despite doubts concerning her taking OC, the patient’s age (32 years old) and lack of other, above-mentioned risk factors and also atherosclerosis allow OC to be ruled out as a possible cause of ICA and MCA thrombosis.

The above-mentioned exclusions and the circumstances preceding the disease established in the course of the investigation indicate the traumatic background of the ICA thrombosis in both cases.

The CT and CT angiography performed in the first patient revealed the presence of a foreign body adjacent to the left ICA on the level of C1, whereas medial translocation and compression of carotid vessels caused by the foreign body were found during the surgical procedure. The histopathological examination revealed features of mechanical arterial injury, namely the rupture of the tunica media with focal haemorrhages into the intima and tunica media. The above-mentioned conditions indicate direct trauma as the cause of thrombosis.

Apart from the arterial occlusion by the thrombus found in the histopathological sections obtained from the second patient, the mechanical injury to the arterial wall in the form of the intima and tunica media rupture were also noted. Such findings and the information obtained during investigation indicate indirect trauma as the cause of arterial thrombosis.

Post-traumatic ICA thrombosis may result from a penetrating (e.g. knife, bullet or other foreign body—splinter, as in case 1) or non-penetrating (blunt) trauma. There are two types of the latter, namely direct ones concerning the neck in the region of the carotid artery, and indirect concerning distant regions, e.g. head [26].

Penetrating neck trauma caused by the above-mentioned tools, leads to arterial wall injury in the form of contusion or vessel wall dissection with the formation of intramural haematoma, outer arterial layer damage (adventitia) and the formation of pseudoaneurysm or segmental vessel spasm [5]. Suppurative inflammation may also develop in the periarterial space, should the patient survive long enough after the trauma. The above-mentioned primary vessel wall injuries, associated with an injury limited to at least endothelium and intima, predispose to the development of thrombosis with subsequent vessel occlusion and ischemic stroke.

The ICA segment localised a few centimetres over the CCA bifurcation is the vessel segment most commonly injured in the course of non-penetrating (blunt) ICA traumas, both direct and indirect. An injury concerning the ICA segment below the skull basis is significantly less frequent. They result in the rupture of the intima and/or tunica media, with the possible formation of intramural haematoma, i.e. a condition called dissecting aneurysm. The thrombotic occlusion of the vessel lumen occurs a few hours or even days or weeks after the trauma [5, 27, 28]. The developing neurological symptoms of ischemic cerebral damage confirm the diagnosis. Six to ten percent of patients manifest such symptoms within the first hour after trauma, another 50 % after 10 h and in 57–73 % of cases the asymptomatic period lasts more than 24 h. Only 17–35 % of such patients develop ischemic symptoms after such a period [8].

The above-mentioned facts indicate that in some cases of traumatic ICA thrombosis there is a variably long asymptomatic period between the trauma and the manifestation of neurological symptoms of ischemic brain damage. Some authors compare it to “*intervallum lucidum”* occurring in patients with epi- and/or subdural haematoma. It results from thrombus propagation within the damaged vessel and may hamper the process of establishing the causal connection between the trauma and the ICA thrombosis, especially, as according to some authors, the process itself may last up to several years [8, 9, 27], which in our opinion is hardly probable.

According to Unterharnscheidt [26] traumatic ICA thrombosis may result from: (1) direct neck trauma (e.g. boxers); (2) direct head trauma (including insignificant) with force transfer to the neck region; (3) oral cavity trauma; (4) fractures of the neuro- and splanchnocranium bones; (5) whiplash injuries; (6) strangling or other manual procedures in the carotid artery region (e.g. massage) and (7) compression by seatbelt in the course of road traffic accidents.

Non-penetrating ICA injuries result from blunt head and/or neck trauma. A head trauma with a violent and powerful head rotation and hyperextension of the cervical spine results in some topographic alterations, including the ICA course. It may lead to ICA compression by the transverse processes of C3 or C1–C2 vertebral bodies and predisposes to thrombosis due to the secondary rupture of intima and tunica media by an intact adventitia. ICA thrombosis may result from neck trauma, as well. By certain head positions, in which the artery is covered only with skin and fascia, it would be pressed against the C1 and C2 or transverse processes of C3–C5 with secondary damage to intima, and even tunica media. In both of the discussed cases the thrombosis could have been caused by a segmental artery spasm on the injury site, which is impossible to establish during a postmortem examination [26].

In 1974 Crissey and Bernstein [27] identified four basic mechanisms of ICA injuries, and thus four types of damage. Type I damage results from a direct blow to the neck and most commonly concerns older patients, frequently having advanced ICA atherosclerosis. A similar mechanism occurs by a sudden powerful hyperflexion of the cervical spine with a compression of the ICA between the mandible and spine. Such an injury is observed in e.g. motorcyclists. Type II ICA damage is caused by the hyperextension of the cervical spine with a head and neck rotation in the opposite direction. Such a mechanism results in injury to the ICA segment that is overstretched over the lateral masses of the first two cervical vertebrae. The discussed mechanisms of ICA injuries, apart from a direct blow to its region, are most commonly observed in motorcyclists participating in road traffic accidents. Type III ICA damage is most commonly observed in children and accompanies the intraoral trauma caused by a foreign body e.g. by falling on a hand-held or more frequently mouth-held object, e.g. pencil. Type IV ICA damage accompanies fractures of the skull basis.

## Conclusions

The establishment of the causal connection between neck and/or head trauma and ICA thrombosis and its cerebral consequences requires the exclusion of non-traumatic factors in the first line—disease-associated, being the most common cause of thrombosis. This is very important as such trauma is most frequently a result of a criminal offence and brings the perpetrator to criminal and civil liability.

The establishment of a causal connection may be problematic or even impossible in victims who apart from traumatic injuries suffer from diseases or factors predisposing to thrombosis.
